# Collaborative Allocation and Optimization of Path Planning for Static and Mobile Sensors in Hybrid Sensor Networks for Environment Monitoring and Anomaly Search

**DOI:** 10.3390/s21237867

**Published:** 2021-11-26

**Authors:** Yanjie Guo, Zhaoyi Xu, Joseph Saleh

**Affiliations:** School of Aerospace Engineering, Georgia Institute of Technology, Atlanta, GA 30332, USA; guoyanjie@gatech.edu (Y.G.); zxu328@gatech.edu (Z.X.)

**Keywords:** hybrid sensor network, environmental monitoring, multi-agent system, sensor allocation, path planning

## Abstract

In this study, a novel collaborative method is developed to optimize hybrid sensor networks (HSN) for environmental monitoring and anomaly search tasks. A weighted Gaussian coverage method hs been designed for static sensor allocation, and the Active Monitoring and Anomaly Search System method is adapted to mobile sensor path planning. To validate the network performance, a simulation environment has been developed for fire search and detection with dynamic temperature field and non-uniform fire probability distribution. The performance metrics adopted are the detection time lag, source localization uncertainty, and state estimation error. Computational experiments are conducted to evaluate the performance of HSNs. The results demonstrate that the optimal collaborative deployment strategy allocates static sensors at high-risk locations and directs mobile sensors to patrol the remaining low-risk areas. The results also identify the conditions under which HSNs significantly outperform either only static or only mobile sensor networks in terms of the monitoring performance metrics.

## 1. Introduction

Sensor networks are the focus of an increasingly vigorous research area with extensive applications in environmental monitoring and anomaly detection [1,2], in maritime search and rescue for example [3], in target tracking [4], and within the broad context of the Internet of Thing (IoT) [5,6,7,8]. With the recent development in low-cost robots, more applications consider the adoption of hybrid sensor networks (HSNs) which exploit mobile sensors in conjunction with static sensors that are smartly distributed across the environment to be monitored [9,10,11,12,13,14,15]. Traditional static sensors are low-cost but cannot be relocated once deployed. Recently developed mobile sensors are more expensive, but their mobility allows for a dynamic coverage area for the smart adjustment of monitoring and search patterns as new risks or priorities emerge. When carefully designed, HSNs can outperform purely static sensor networks via a collaboration between the two types of sensors. This observation prompts some general questions, for example, how should the static sensors be allocated, and the path planning of the mobile sensors formulated to achieve a collaborative optimal monitoring performance? What is the optimal sensor portfolio mix (of static and mobile sensors) and what is it contingent upon? These are some of the questions addressed in this work.

There are three challenges in the current research on HSNs. First, the deployment strategy of HSN does not fully consider the collaboration between static and mobile sensors, and optimization has not yet been investigated for the portfolio mix of the two types of sensors under cost constraints. Second, the currently adopted environment models are fairly basic with static fields, uniform risk distribution, and a disk-shaped sensing model. Real environments that require monitoring are more complex and rarely conform to these simple assumptions. Third, general performance metrics, such as coverage percentage and communication efficiency, are generally adopted to evaluate the sensor networks. What is missing are more direct assessment in terms of the optimal monitoring performance for specific applications, such as anomaly detection time. A more detailed discussion of the related work is provided in Section 2.

The work focuses on the design and optimization of collaborative hybrid sensor networks (HSNs). This work addresses the aforementioned open issues for general sensor networks. More specifically, a novel collaborative method is developed and validated to optimize HSNs for environmental monitoring and anomaly search tasks. A weighted Gaussian coverage (WGC) method has been designed for the static sensor allocation, and the Active environmental Monitoring and Anomaly Search System [16] has been adapted for the mobile sensor path planning. While the deployment method is application-agnostic and can be used for a broad range of environmental monitoring tasks, a task of fire search in a multi-room apartment is chosen as the main application to demonstrate its performance. To validate the network performance, a realistic simulation environment was developed, with a dynamic temperature field and a (spatially) non-uniform fire probability distribution. A physical sensing model has also been adopted based on temperature fire detectors [17]. The performance of different sensor networks is evaluated by the ultimate monitoring performance (e.g., detection time lag) instead of indirect metrics (e.g., area coverage).

The results (1) demonstrate that the optimal collaborative deployment strategy allocates the static sensors at high-risk locations and directs the mobile sensors to patrol the remaining low-risk areas; (2) identify a set of conditions under which HSNs significantly outperform purely static and purely mobile sensor networks in terms of the ultimate monitoring performance; (3) establish how the cost constraints and mobile sensor speed affect the optimal sensor portfolio.

The main contributions of this work are the following:

1. A general optimization problem is formulated for HSNs with static and mobile sensors and solved to identify the optimal portfolio mix and its main drivers.
2. A collaborative deployment strategy of HSN is developed to improve the monitoring performance in complex environments with obstacles and non-uniform risk distribution.
3. A realistic simulation environment is built to provide a more direct assessment of the ultimate performance objectives of monitoring and anomaly search tasks.

The remainder of the article is organized as follows. A literature review is provided in Section 2. The HSN optimization problem formulation and the research objectives are introduced in Section 3. The details of the collaborative sensor deployment method are provided in Section 4. The simulation environment and performance metrics used to evaluate and validate the deployment method are discussed in Section 5. The computational results and discussion are presented in Section 6. Finally, in Section 7 provides the conclusion.

## 2. Related Work

The design of HSNs can be naturally classified into two parts: a static sensor allocation part and a mobile sensor path planning part. The questions in the introduction indicated the need for a collaborative combination of these two parts. Most previous works considered the collaboration between mobile sensors only [18,19], or just used mobile sensors to assist a group of pre-allocated static sensor [20,21]. For example, Popescu et al. [18] examined the collaboration between unmanned aerial vehicles (UAVs). Sun et al. [21] focused on the collaboration between mobile sensors for spatiotemporal coverage. Freitas et al. [20] adopted UAVs to review an area of interest after an alarm had been triggered by the static sensors. While these studies make important contributions, they do not consider the joint static sensor allocation and mobile sensor path planning problems simultaneously, or the potential collaboration between them. 

Another important aspect that has not received adequate attention in the literature is the environment model. Previous works generally assumed a simple environment and focused on sensor networks. For example, refs. [22,23] investigated the search and localization problem of a stationary target with static measurement field. Research in [9,10,24,25] assumed a static environment with uniform event occurrence probability distribution. The work reported in [10,11,24,26,27] adopted the commonly used disk-shaped Boolean or probabilistic sensing model [28]. However, these simplified environment models may not reflect the dynamics in real applications. For instance, the temperature field is usually unsteady in an environment [29]; the seismic hazard map is highly non-uniform [30]; and the measurements of gas sensors are affected by dynamic airflow [31]. Recent studies [32,33] have highlighted the need to examine more complex environments with time-varying environmental fields, non-uniform point-of-interest or risk maps, and physical sensing models to meaningfully assess the performance of monitoring sensor networks.

To evaluate the performance of monitoring sensor networks, previous works proposed several metrics, which can be divided into three general groups, as follows: sensor coverage, network connectivity, and energy efficiency [33]. For example, Chakraborty et al. [12] proposed a coverage-reliability index for mobile sensor networks to measure the coverage area with reliable data delivery. Njoya et al. [26] addressed the sensor placement problem with guaranteed connectivity. Additionally, other researchers have focused on energy-efficient target-tracking strategies for mobile sensor networks [34,35]. These metrics are important for the general design of sensor networks for several reasons: the overall sensor coverage impacts the monitoring performance of a network; the connectivity of sensors affects the information/data transmission within the network; and the energy efficiency affects the lifetime of a network. While they are thought to be suitable for the general design of sensing networks, these metrics are indirect and inadequate to evaluate the ultimate monitoring performance of specific applications. For instance, detection time lag for fire detection and source localization for avalanche search and rescue are the ultimate and direct performance metrics of monitoring networks for these particular applications [23]. Using only general, indirect metrics is myopic and misses the important final step for specific applications, as will be discussed and illustrated later in this work.

To address these three challenges, in this work, first, a general optimization problem for HSN with static and mobile sensors is formulated. Second, a collaborative HSN deployment strategy is developed to improve the monitoring performance in complex environments with obstacles and non-uniform risk distribution. Third, a realistic simulation environment is built to provide a direct assessment of the ultimate objectives of specific monitoring and anomaly search tasks.

## 3. Problem Formulation and Assumptions

In this section, a general optimization problem for HSN is formulated. Curious readers may refer to Section 5 for a specific scenario to better understand the general problem.

The objective of this work can be succinctly expressed as follows: to design and optimize the performance of a hybrid sensor network (HSN) by collaborative static sensor allocation and mobile sensor path planning. Before elaborating on the specific research objectives, the main design parameters considered (Table 1) and assumptions are first introduced to facilitate the subsequent discussion.

First, the cost parameters are considered. The total budget for the sensor network is set as C. The cost of a static sensor is defined as the unit cost, and the cost of a mobile sensor as γ, which illustrates the cost ratio between them. Since the focus in this work is not on the detailed cost structure, e.g., design cost, manufacturing cost, integration, testing, and operational costs, a constant total cost is used to account for all costs. Note that while some cost might seem to be time-varying (e.g., running cost for mobile sensors), this issue can be worked around by setting a fixed expected working life for the sensors (e.g., 50 thousand hours for a mobile sensor) and calculating an approximate constant total cost. Each mobile sensor is assumed to consist of a static sensor mounted on a moving platform. Thus, the cost ratio is larger than one, γ>1. In practice, the mobile element usually consists of a larger suite of sensing devices. In this work, it is assumed that both sensors have the same sensing device so that the mobility effect can be studied independently.

Second, the sensor portfolio parameters are considered. The number of static and mobile sensors in the sensor network are defined as $n_{f}$ and, respectively. The total number of sensors in the network is $N_{tot}=n_{f}+n_{a}$, and the sensor network total cost $C_{tot}$ can be calculated by Equation (1):

(1) $$C_{tot}=n_{f}\cdot \left(\cos t\;\mathrm{of}\;a\;\mathrm{static}\;\mathrm{sensor}\right)+n_{a}\cdot \left(\cos t\;\mathrm{of}\;a\;\mathrm{mobile}\;\mathrm{sensor}\right)=n_{f}+\gamma n_{a}$$

Note that for environments with an arbitrary size, sensor density is a more appropriate parameter to describe the quantity of sensors. Since the focus here is on one environment with a fixed size, sensor density is considered to be the same as sensor number, and they are used interchangeably in the later discussion. Given the linearity of Equation (1), the iso-cost curve in the $n_{f}$-$n_{a}$ space is represented by a straight line. This observation will be useful later in the analysis in Section 6. Since only two types of sensors are considered, the sensor portfolio can be characterized by the proportion of either type. Without loss of generality, the proportion of the mobile sensors among all sensors $\beta =n_{a}/N_{tot}\in \left[0,1\right]$, has been selected and referred to as the portfolio mix hereafter. For example, β=0 indicates a purely static sensor network; β=1 a purely mobile one; and $\beta \in \left(0,1\right)$ a hybrid sensor network. The sensor network cost $C_{tot}$ can be rewritten using β in Equation (2):

(2) $$C_{tot}=\left(1-\beta \right)N_{tot}+\gamma \beta N_{tot}=\left(1-\beta +\gamma \beta \right)N_{tot}$$

Having established these design parameters, the problem formulation is presented. Previous works have generally formulated the design of sensor networks as optimization problems [13,32,36]. In this work, the problem is formulated as a cost-constrained optimization problem, as shown in Equation (3):

(3) $$\begin{array}{cc} \underset{n_{f},n_{a},\Phi }{\max} & \mathrm{performance}\left(\Phi \left(n_{f},n_{a}\right)\right)\\ \mathrm{subject}\;\mathrm{to} & C_{tot}=n_{f}+\gamma n_{a}\leq C \end{array}$$

A sensor portfolio $\left(n_{f},n_{a}\right)$ and a collaborative sensor deployment method Φ need to be formulated to maximize certain performance metrics and guarantee that the total cost is within the budget $C_{tot}\leq C$. Note the following remarks on Equation (3). First, the performance is a general objective function to be optimized. For example, it can minimize the detection time lag for anomaly detection or maximize the localization accuracy for target search. A detailed discussion of the performance metrics is provided in Section 5.2. Second, Φ is a dummy variable for the deployment method that includes static sensor allocation and mobile sensor path planning for HSNs. Third, the energy constraints on the sensors are excluded because, compared with a typical single-cycle battery runtime of several hours or more, the total runtime for the example monitoring task is significantly shorter, as will be illustrated later. 

Next, Equation (3) has been simplified to an equivalent optimization problem. Since the optimal performance is achieved by using the maximum budget  C, the inequality constraint is always active. If the sensor portfolio mix β is given, Equation (2) can be used to compute the total number of sensors by Equation (4):

(4) $$N_{tot}=|\frac{C}{1-\beta +\gamma \beta }|,$$

where the floor function ᒪ⋅ᒧ gives the greatest integer less than or equal to its argument. Note that the denominator in Equation (4) does not disappear and this expression is well-defined. In other words, given the cost parameters $\left(C,\gamma \right)$, the total number of sensors $N_{tot}$ can be calculated by finding the optimal portfolio mix β. Since the sensor portfolio $\left(n_{f},n_{a}\right)$ can be obtained from $\left(N_{tot},\beta \right)$, the design variables are switched to β and Φ. Equation (3) is then simplified to an equivalent problem as shown in Equation (5):

(5) $$\begin{array}{cc} \underset{\beta \in \left[0,1\right],\Phi }{\max} & \mathrm{performance}\left(\Phi \left(\beta ;C,\gamma \right)\right) \end{array}$$

The optimal portfolio mix β and a collaborative deployment method Φ should be determined given the cost parameters $\left(C,\gamma \right)$. This problem is solved in a divide-and-conquer manner. First, a collaborative sensor deployment method Φ is devised given the sensor portfolio β, i.e., the sensor numbers $n_{f}$ and $n_{a}$ are first given. Second, with the deployment method Φ, simulations are conducted for different sensor portfolio β to identify the optimal sensor. Note that for practical use with one given cost condition, only a line search algorithm along the corresponding iso-cost curve is needed. By varying the cost parameters, a Pareto front is obtained for the network cost-performance multi-objective optimization problem.

There are three research objectives. First, to devise a collaborative sensor deployment method Φ with both static sensor allocation and mobile sensor path planning given the sensor portfolio mix β (O1). Previous works have either studied them separately or only focused on how mobile sensors can assist pre-allocated static sensors [20,37]. The present work will demonstrate the importance of the collaborative allocation of a static sensor in ways that can facilitate mobile sensors’ path planning and exploration. Second, to identify the optimal sensor portfolio β for different cost parameters $\left(C,\gamma \right)$ (O2). Most previous works only consider HSNs with pre-set (not modifiable) sensor portfolio [10,38]. In this work, a cost-performance analysis is conducted for different sensor portfolios, and the optimal portfolio mix is determined under various cost conditions. Third, to investigate the effect of the mobile sensor speed on the optimal portfolio mix (O3). To address these questions, the HSN deployment method Φ is introduced in the next section.

## 4. A Collaborative Deployment Method for Hybrid Sensor Networks

The design of the deployment method Φ is formulated as a sub-problem of Equation (5):

(6) $$\begin{array}{cc} \underset{\Phi }{\max} & \mathrm{performance}\left(\Phi \left(\beta ;C,\gamma \right)\right) \end{array}$$

Note that the focus here is on the first research objective (O1). The sensor portfolio β is assumed and the deployment strategy Φ is considered as the design target. Simulations with different sensor portfolios is conducted later in Section 6.2 to identify the optimal sensor portfolios with different cost conditions (O2). The aim is to optimize the network performance through collaboration and shared measurements between the static and mobile sensors. Next, a high-level overview of the deployment method is provided, and its technical details are discussed in the following subsections.

### 4.1. Overview

**Figure 1:**
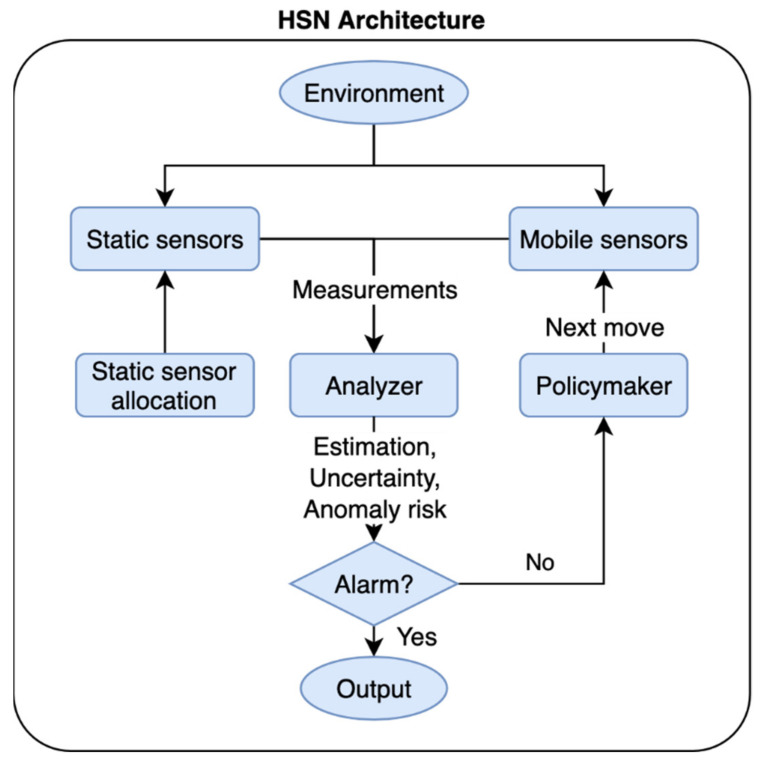
The architecture of hybrid sensor network deployment method.

The HSN deployment method consists of two parts, static sensor allocation (SSA) and mobile sensor path planning (MSPP). The high-level HSN architecture is shown in Figure 1.

Most realistic environments have a non-uniform risk map. For example, cooking in the kitchen, electric heaters in the bedrooms, and dryers (wherever they are placed) exhibit a higher risk of fire. With this observation, the collaboration between static and mobile sensors is achieved by allocating the static sensors at high-risk locations and utilizing the mobile ones to patrol the rest of the area. In the absence of a risk map or for an unknown environment, the default method is to use a uniform risk map, which is a special feature of the more general solution. The following discussion addresses HSN deployment problems with a uniform and non-uniform risk map.

A weighted Gaussian coverage (WGC) metric is designed to solve the SSA problem. The active monitoring and anomaly search system [16] is adapted to solve the MSPP problem online. There are two main components in the MSPP process, an analyzer and a policymaker. At each time step, the analyzer takes the raw measurements from both the static and mobile sensors and extracts the relevant information, including full state estimation of the environment, estimation uncertainty level, and the risk or likelihood of an anomaly at particular locations. This information represents a high-level understanding of the environment and is used for anomaly detection and MSPP. If the state estimation or likelihood of anomaly breaches a predefined threshold, an alarm is triggered. Otherwise, the policymaker determines the next optimal moves for the mobile sensors based on a Markov decision process (MDP) model. The policymaker formulates a parameterized reward function based on the extracted information from the analyzer to solve the MSPP problem, using a Dynamic Value Iteration (DVI) method developed in [16]. Next, the technical details of each component are discussed.

### 4.2. Static Sensor Allocation with Weighted Gaussian Coverage

In this subsection, the first step of the deployment method is discussed, which is static sensors allocation. Previous works studied the uniform sensor allocation problem [39,40]. This work considers a more general problem of distributing sensors to cover high-interest areas according to a non-uniform risk map, should it be available, and a default uniform map if not. The general problem can be formulated as a discrete optimization problem, given by Equation (7):

(7) $$\underset{s}{\max}U\left(s\right)\;s=\left\{s_{1},s_{2},\ldots ,s_{n}\right\},\;s_{i}\in L,\;i=1,2,\ldots ,n<\left|L\right|$$

where L is a finite set of all possible sensor locations (through discretizing the environment into grids for example). n locations need to be chosen from L to maximize a metric of sensor coverage U. The choice of these n locations is denoted as $s=\left\{s_{1},s_{2},\ldots ,s_{n}\right\}$. Note that $n<\left|L\right|$, and this can be guaranteed by discretizing the environment into smaller grids. 

The sensor coverage U needs to reflect the ultimate monitoring performance. The most commonly used metric, area coverage percentage, has some drawbacks: it requires a fixed coverage radius for each sensor; it is not suitable for non-uniform risk distribution; and it does not consider the dynamics of the environment. Such is the case for fire detection for example, as it takes time for the heat and smoke to transport to the sensor location. In other words, the detection area of the sensors depends on time, the dynamics of the environment, and the geometry, among other things. 

To address these drawbacks, a weighted Gaussian coverage (WGC) is proposed as shown in Equations (8)–(10):

(8) $$G\left(l;s_{i},\sigma \right)=\exp\left(-\frac{d{\left(l,s_{i}\right)}^{2}}{2\sigma^{2}}\right),\;\;l,s_{i}\in L$$

(9) $$K\left(l\right)=\underset{s_{i}\in s}{\max}G\left(l;s_{i},\sigma \right)$$

(10) $$U\left(s\right)=\underset{l\in L}{\sum }w\left(l\right)\cdot K\left(l\right)$$

**Figure 2:**
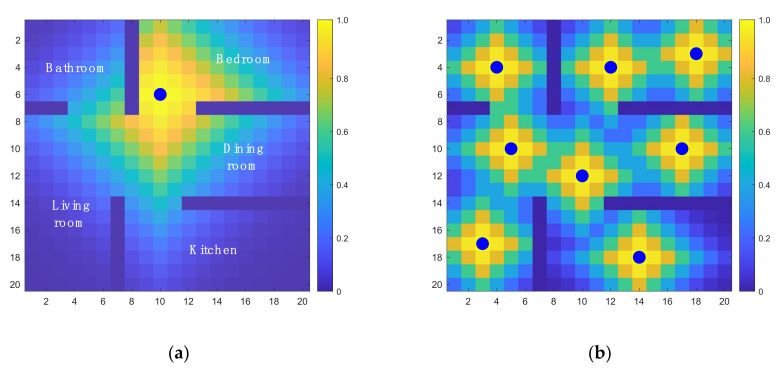
Example kernel and coverage index functions for sensors in a $20\times 20\;m^{2}$ apartment. (**a**) Kernel function of one random sensor; (**b**) Coverage index function with 8 sensors. The blue dots represent sensor locations. Note that in (**a**), the kernel function in the bathroom is different from one without the wall.

First, at each sensor location $s_{i}$, a Gaussian kernel $G\left(l;s_{i},\sigma \right)$ with standard derivation σ is included to represent its capture area, as shown in Equation (8). This kernel G generally defines well another location l can be monitored by a sensor located at $s_{i}$. Note that the distance function $d\left(l,s_{i}\right)$ represents the travel distance between these two locations accounting for potential obstacles in the environment. The travel distance can be calculated by path search algorithms such as A* [41]. Figure 2a shows an example kernel in an environment with obstacles. This choice of using travel distance in the kernel reflects in part some monitoring performance metrics such as detection time lag and distance between sensor and anomaly to be detected. Second, at each location l, the maximum kernel value among all sensors is taken as an index of coverage $K\left(l\right)$ to indicate how well this location can be sensed by all sensors, as shown in Equation (9). Finally, the weighted sum of the coverage index over the entire space is calculated according to a predefined spatial risk probability $w\left(l\right)$, as shown in Equation (10). When the environment is unknown or has a uniform risk map, a uniform weight is set. For a non-uniform risk map, the WGC method allocates static sensors closer to the high-risk locations. Figure 2b shows an example allocation of eight sensors in the same environment.

### 4.3. Mobile Sensor Path Planning

After the static sensors are allocated, the active monitoring and anomaly search system (AMASS) [16] is adapted to the MSPP problem. AMASS is based on a Markov decision process model. The objective of AMASS is to detect anomalies in an environment in a timely manner. The ideal response for the mobile sensors are identified by maximizing the cumulative reward given by a parameterized function formulated with extracted information from the raw measurements. In this subsection, important technical details of the extracted information, the reward function, and the multi-agent system are provided for the reader’s convenience. More details can be found in [16].

As shown in Figure 1, three pieces of information are extracted from the raw measurements, the full state estimation E, the estimation uncertainty U, and the conditional risk of anomaly ψ at each location. Assuming the dynamics of the environment are known, a Kalman filter or a nonlinear filter (e.g., particle filter) can be built depending on the dynamics to obtain the full state estimation E and the covariance matrix P. If the dynamics of the environment are unknown, these two pieces of information can be obtained by a Gaussian process regression with a potentially larger error. The diagonal of the covariance matrix P is the variance of the estimation error at each state. Assuming a Gaussian distribution, a 95% uncertainty U can be computed with Equation (11):

(11) $$U=2\sqrt{diag\left(P\right)}$$

The last piece of information, the anomaly risk ψ, is obtained by Equation (12):

(12) $$\psi =1-{\left(1-\phi_{A}\right)}^{\Delta t_{l}},$$

where $\Delta t_{l}$ is the amount of time passed since the most recent visit at location l, and $\phi_{A}$ is the probability of having anomalies over a unit time period. This information helps to monitor the time elapsed between visits for each location (for $\Delta t_{l}$). The longer this period, the higher the risk of an anomaly at this location.

The reward function is formulated by the extracted information with a balance of exploitation and exploration:

(13) $$R=\tilde{E}+\epsilon_{U}U+\epsilon_{\psi }\psi ,$$

where $\tilde{E}$ is a generalized exploitation index based on the estimation and $\epsilon_{U}$ and $\epsilon_{\psi }$ are two parameters controlling the exploration rate. On the one hand, high reward is provided at locations where the estimation is unusual. $\tilde{E}$ can be expressed with a simple linear relationship $\tilde{E}=\left|E-E_{0}\right|/\Delta E$, where $E_{0}$ is the nominal value for the estimation and ΔE controls the off-nominal tolerance. Note that this relationship can also be one-sided depending on the anomaly (e.g., fire event only happens when the temperature measurement is unusually high). On the other hand, high reward is provided at locations where the estimation uncertainty is high and has not been revisited for a long time.

A critical problem for a system of mobile sensors is the possibility of multiple sensors being in the same location. Crowded mobile sensors result in system inefficiency and have higher risk of collisions. The computational complexity of the global optimum path planning for a multi-agent system increases exponentially with the number of agents [42]. Practically, decentralized approaches are adopted to find a local sub-optimum given the available computational power. In this work, for each mobile sensor, the reward it can obtain is scaled down around the other sensors to prevent it from moving towards them. The scaling effect is a similar Gaussian kernel given by:

(14) $$ker_{i}\left(l;s_{i},\sigma \right)=1-\exp\left(-\frac{d{\left(l,s_{i}\right)}^{2}}{2\sigma^{2}}\right),$$

where $s_{i}$ is the location of a mobile sensor i, σ is a parameter controlling the kernel size. σ is set to be proportional to the average sensor distance with a factor p, $\sigma =p\cdot {\left(\frac{total\;area}{n_{f}+n_{a}}\right)}^{1/2}$. This kernel maintains the reward that is far from the sensor and scales down the reward that is close to it. The adjusted reward function for mobile sensor i is derived by applying this kernel to all the other sensors and taking the minimum value:

(15) $$R_{i}\left(l\right)=R\left(l\right)\cdot \min_{j\neq i}ker_{j}\left(l;s_{j},\sigma \right)$$

The dynamic value iteration method [16] is used to solve for the next optimal movement for each mobile sensor based on this adjusted reward function. This can be performed either in a central system with parallel computing or be distributed to each sensor to compute locally.

This concludes our discussion on the collaborative HSN deployment method by combining the static sensor allocation and mobile sensor path planning. In the next section, the simulation environment developed for the application and the performance metrics used for the sensor networks are discussed.

## 5. Application-Specific Simulation Environment and Performance Metrics

In Section 3, the problem statement is defined for a general monitoring task with an arbitrary performance metric as the objective function. In this section, the discussion is narrowed down to the specific application of fire searches, in a simulation environment with a dynamic temperature field. The simulation environment and the specific performance metrics used hereafter are introduced next.

### 5.1. Simulation Environment

**Figure 3:**
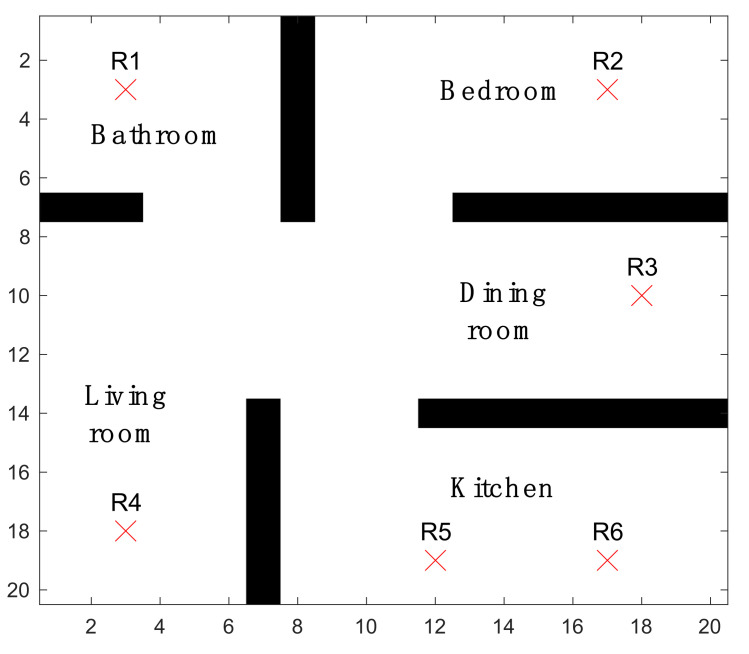
Example environment of a $20\times 20{\;m}^{2}$ apartment for temperature monitoring and fire search and detection. The red crosses indicate locations with higher fire risks (R1–R6).

The application is for temperature monitoring and fire detection in a 2D multi-room apartment. The floorplan of the apartment is shown in Figure 3. A $20\times 20{\;m}^{2}$ apartment is discretized into $1\;m^{2}$ grids for potential sensor locations. The mobile sensors can move to the four neighboring grids or stay at each time step. Note that although the current environment is a regular apartment, the deployment method and the network monitoring performance is scalable to the environment size as long as the sensor density is the same. For example, 10 sensors in an $10\times 10{\;m}^{2}$ area will have a similar performance to 40 sensors in a $20\times 20{\;m}^{2}$ area.

There are three key characteristics of this application. First, the temperature field is dynamic. The purpose of building the simulation environment is to obtain a dynamic temperature field inside a room when a fire event occurs. A conduction heat propagation process is adopted to simulate realistic indoor conditions [43]. A detailed dynamic model is presented in Appendix A. Second, fire risk is non-uniform in this room. This conforms with a previous observation that some locations have higher risks of fire. For example, cooking in the kitchen and electric heaters in the bedrooms exhibit a higher fire risk. The high-risk locations are marked with red crosses in Figure 3. Third, a physical sensing model for the fire detection process is adopted instead of the commonly used disk-shaped model with a predefined sensing radius. A fire event is detected according to the measured temperature. The sensor selected for the computational experiments is a pointwise temperature sensor with alarm threshold at 47 ℃ [17]. The sensors trigger a fire alarm when the measured temperature breaches this threshold.

Next, the performance metrics that are used as the objective functions are discussed.

### 5.2. Performance Metrics

As noted in the introduction, previous sensor network deployment methods focused on improving sensor coverage, network connectivity, and energy efficiency. However, for a given application in a complex environment, the aforementioned metrics do not necessarily reflect the ultimate aim of the monitoring tasks, such as detection time and state estimation error. In this work, the three performance metrics listed in Table 2 are considered as the ultimate objective for the fire detection application [16].

First, detection time lag represents the temporal sensitivity of the sensor network. Early detections grant people more reaction time. Second, alarm distance indicates the spatial uncertainty of the anomaly source when it is detected. The reasoning behind using this metric is that when an alarm is triggered, the source of the anomaly is often uncertain. For example, it took several months to localize the source of a leak on-board the International Space Station after its detection [44]. It will be easier and faster to perform the subsequent search and intervention if this spatial uncertainty is reduced. Third, the estimation error reflects the general monitoring performance over the whole space. The median error is adopted because the estimation can present a significant error in a small region around the anomaly source. This deviance will affect both the mean and the maximum estimation error, whereas the median error is less biased and more robust. Therefore, the entire distribution of the state estimation error is more ambitious and computationally challenging. It is left as a potential avenue for future work.

Note that calculating these metrics requires the ground truth provided by the simulation environment. These metrics serve as an evaluation of the sensor network for the testing purpose.

Next, these three metrics are used as the objective functions to evaluate the performance of the sensor networks.

## 6. Computational Experiments: Results and Discussion

In this section, the results of the computational experiments are discussed in light of the three research objectives O1–O3 discussed in Section 3. An example simulation result of one sensor portfolio is provided to illustrate the nature of the collaborative hybrid sensor method (O1). To identify the optimal sensor portfolio (O2), the network performance is evaluated for multiple portfolio candidates under various cost conditions. Additional experiments are conducted to examine the impact of the mobile sensor on the network performance and the optimal sensor portfolio (O3).

### 6.1. Example Simulation Result of One Sensor Portfolio Candidate

**Figure 4:**
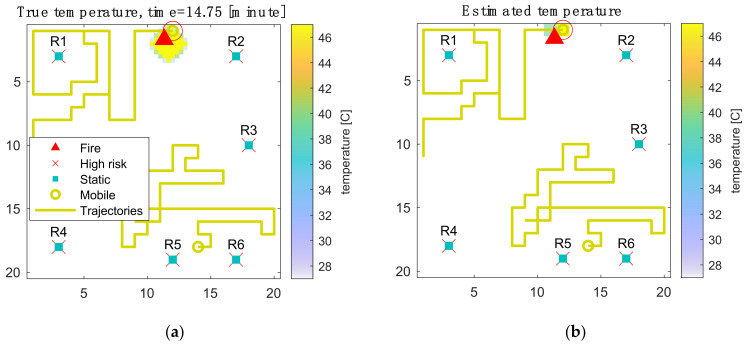

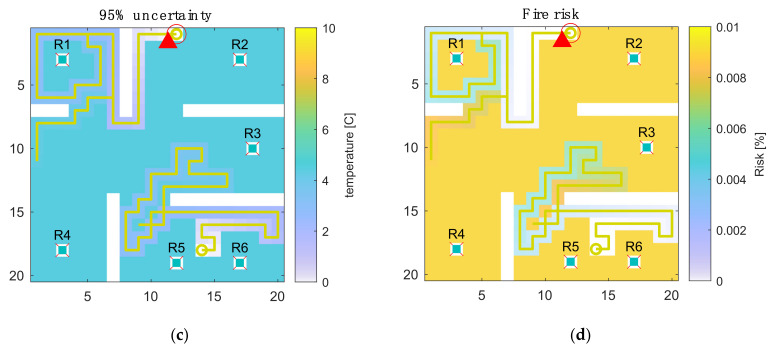
The final snapshot of one example simulation of 6 static sensors and 2 mobile sensors. The four maps show (**a**) the true temperature; (**b**) the estimated temperature; (**c**) the 95% uncertainty of the estimation; and (**d**) the estimated fire risk.

In this example, 6 static sensors and 2 mobile sensors with a moving speed of 6.67 cm/s are deployed for illustrative purposes. At the start of each simulation, an initial fire location is randomly selected according to the fire risk map. The simulation continues until the fire is detected by one of the sensors. For the robustness of the results, the simulation is repeated one hundred times with different initial fire locations. The weighted mean performance according to the fire risks is used for the comparison. A snapshot obtained at the end of the simulation is shown in Figure 4.

In this example, all six static sensors are located at high-risk locations. The sensors are allocated this way to achieve the highest weighted Gaussian coverage (WGC). The two mobile sensors are set to avoid each other as well as the static sensors to explore the environment more efficiently. This simple result illustrates how the static sensors can be used to monitor fixed high-risk locations, and the mobile sensors can patrol a larger area that has a dynamic risk uncertainty. The fire is detected after 14 min because the heat transport is relatively slow under the defined conditions. This level of performance is compared with other sensor portfolios in the following experiments and optimal portfolios are identified under various cost conditions.

### 6.2. Cost-Performance Tradeoff Analysis and Sensor Portfolio

**Figure 5:**
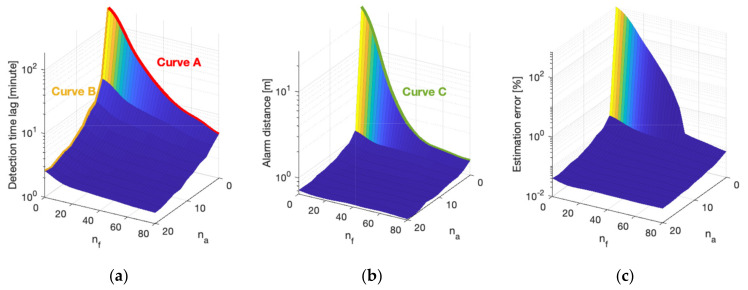
Weighted mean performance for different sensor configurations: (**a**) Detection time lag; (**b**) Alarm distance; (**c**) Estimation error.

The optimization of the sensor portfolio is a central problem for HSNs with cost constraint. It is important to determine the benefit of adding each type of sensor and to balance the tradeoffs between them. A performance saturation point can be reached when adding sensors of the same type beyond a certain point. The performance of a large combination of different sensor numbers $\left(n_{f},n_{a}\right)$ is analyzed to understand this performance landscape. Additionally, the marginal improvement of different sensor portfolios is investigated. The moving speed for the mobile sensors is set to be 5 cm/s, and the sensor numbers are varied for $n_{f}\in \left[0,80\right]$ and $n_{a}\in \left[0,20\right]$. The results are provided in Figure 5.

Several important results are displayed in Figure 5. The most salient are the following:

1. Adding more sensors of any type will improve all performance metrics. However, this improvement is highly non-linear, and it indicates a decreasing marginal benefit with higher sensor density. Consider curve B (mobile sensors) in Figure 5a for example, whereby a proverbial knee in the (performance) curve exists, and it occurs around approximately 3 mobile sensors. The incremental advantage of having a higher sensor density decreases beyond this point. The benefit of an increase from 1 to 3 mobile sensors is significant; in contrast, the benefit of an increase from 18 to 20 is negligible. This asymptotic behavior displayed in Figure 5a reflects that it approaches the point of network performance saturation.
2. The marginal improvement is different for static and mobile sensors (along the $n_{f}$-axis versus the $n_{s}$-axis). Compare, for example, curve A (static sensors) and curve B (mobile sensors) in Figure 5a whereby the detection time lag can be improved from over 100 min to 5 min with 80 static sensors (curve A), while the same level of improvement can be achieved by as few as 8 mobile sensors (curve B). The iso-cost performance of different sensor portfolios will be compared shortly.
3. The same observations apply to all three performance metrics in Figure 5b,c. The results display similar trends, although the exact shapes of the surfaces are different. For example, when comparing the static sensors when $n_{a}=0$ in Figure 5a (curve A) and 5b (curve C), the knee in the curve occurs at a smaller sensor density for alarm distance (around 45 static sensors) than detection time lag (around 60 static sensors). This indicates that despite having 45 static sensors, adding more can still improve the detection time lag considerably, although this does not improve the alarm distance in any meaningful way.

**Figure 6:**
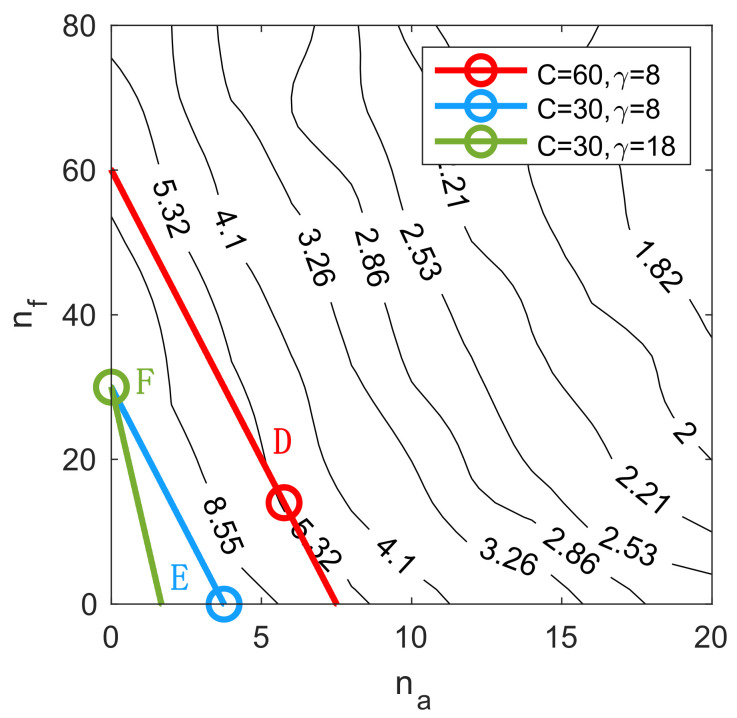
Performance contour of detection time lag with some constant-cost lines. The circles indicate the optimal sensor portfolio with the best iso-cost performance.

The previous results compare the performance of different HSNs and provide some insights into their performance landscape across $\left(n_{f},n_{a}\right)$. Next, the cost constraint is considered, and the optimal sensor portfolio mix $\beta^{*}$ is identified for instances where the total budget is limited. An example contour plot of detection time lag is shown in Figure 6, to illustrate how to identify the optimal sensor portfolios.

As noted in Section 3, the cost constraints are represented by straight lines in the $n_{f}$-$n_{a}$ space according to Equation (1) (which is copied here for the readers’ convenience):

(16) $$C_{tot}=n_{f}+\gamma n_{a}$$

**Figure 7:**
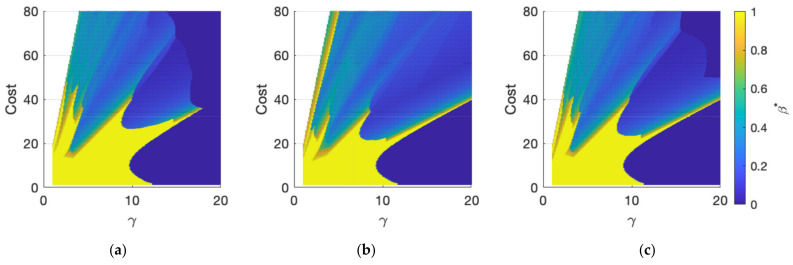
Optimal mobile sensor portfolio mix $\beta^{*}$ for different cost parameters: (**a**) Detection time lag; (**b**) Alarm distance; (**c**) Estimation error. The mobile sensor speed is 5 cm/s.

The intercept (on the $n_{f}$ axis) indicates the sensor cost C, and the slope indicates the cost ratio γ. For example, line D in Figure 6 has a sensor cost of 60 and cost ratio of 8. The optimal iso-cost sensor portfolios, as indicated by the circles in Figure 6, are the points that provide the best performance on a given constant-cost line. Given the necessary condition of constrained optimization [45], the best sensor portfolio is obtained either at the tangent point (if there exists one) between a constant-cost line and a contour level (line D), or on one of the axes (line E and F). Note that points on the axes indicate either static or mobile sensor networks. The optimal portfolio mix $\beta^{*}$ can be easily computed from the best portfolio points $\left(n_{f}^{*},n_{a}^{*}\right)$. Note that for practical use with one given cost condition, the optimal sensor portfolio can be found by a line search algorithm along the corresponding iso-cost line. The results, illustrated in Figure 6, are generalized to a broad range of cost parameters $\left(C,\gamma \right)$ to assess their effect on the optimal sensor porfolio. The results are provided in Figure 7.

The most important result observable in Figure 7 is the existence of certain regions in the C-γ space, where hybrid sensor networks ($0<\beta^{*}<1$) provide the best monitoring performance. The other salient results in Figure 7 are as follows:

1. When γ is large, mobile sensors are more expensive than static sensors. As a result, the optimal portfolio tends to include only static sensor networks with $\beta^{*}=0$ (dark blue areas in Figure 7).
2. When γ is small, mobile sensors are more economical. For a limited range of total cost C, the optimal configuration tends to include only mobile sensor networks with $\beta^{*}=1$. (yellow areas in Figure 7).
3. Outside these two conditions, for small γ and larger C than in (2), the results in Figure 7 indicate that hybrid sensor networks become the optimal configuration. One way of conceptualizing this result is that with larger C, the network density is higher, and as result, either purely static or mobile sensor networks approaches their saturation points. Thus, the incremental improvement from adding the same sensors in the network decreases dramatically. However, adding a different sensor still improves the performance, resulting in $0<\beta^{*}<1$ or a hybrid sensor network as the optimal portfolio mix.

Given the optimal portfolio mix, the performance–cost Pareto fronts can also be derived. The detailed results are discussed in Appendix B. 

This series of experiments demonstrates that hybrid sensor networks (HSNs) outperform purely static and mobile sensor networks under specific cost conditions. The sensor portfolio mix β is a critical parameter that needs to be carefully assessed and optimized to achieve the ideal iso-cost performance. Next, another critical parameter, the mobile sensor speed, is examined, and its impact on the network performance and the optimal portfolio mix is assessed.

### 6.3. Investigating the Impact of Mobile Sensor Speed on the Monitoring Performance

The speed of the mobile sensors is an important parameter that affects the network monitoring performance. The extent of this effect, however, is not negligible and will depend on the sensor density and portfolio mix in a complex manner. In the previous experiments, the mobile sensor speed was held constant at 5 cm/s. In the next series of experiments, the speed is set at (10, 15, and 20) cm/s respectively, and the changes in monitoring performance for different sensor network portfolios are investigated.

**Figure 8:**
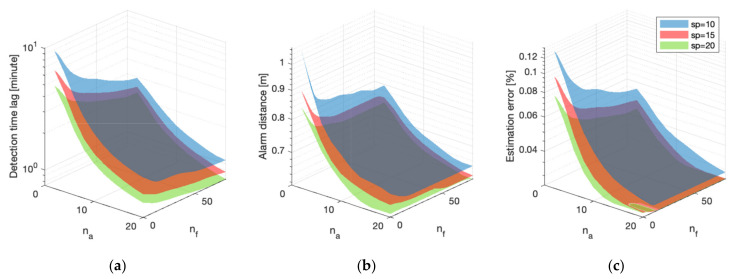
Performance comparison of mobile sensor speed for different sensor portfolios: (**a**) Detection time lag; (**b**) Alarm distance; (**c**) Estimation error. The view angle is different for this plot to show the three surfaces.

The performance surfaces for different mobile sensor speeds are provided in Figure 8. Note that the purely static sensor networks when $n_{a}=0$ are excluded since the moving speed does not affect their performance.

**Figure 9:**
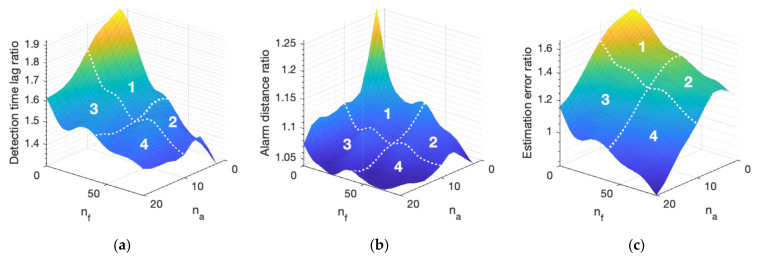
Performance ratio between 10 cm/s and 20 cm/s for different sensor portfolios: (**a**) Detection time lag; (**b**) Alarm distance; (**c**) Estimation error. The $n_{f}$-$n_{a}$ plane is divided into four regions (1–4).

A similar general trend is observed for different mobile sensor speeds, such that higher moving speeds generally result in better monitoring performance across the three metrics. To better visualize this performance differential for different speeds, the performance ratio, for example, between 10 cm/s and 20 cm/s is computed. The results are shown in Figure 9. The performance ratios between the other speeds share a similar trend. The $n_{f}$-$n_{a}$ space is divided into four regions based on the sensor density to facilitate the discussion.

Several observations can be made based on the results in Figure 9. The most salient ones are the following:

1. In region 1, where both static and mobile sensor densities are limited, the performance ratio is the highest among the four regions when the speed is increased from 10 cm/s to 20 cm/s. This is the greatest improvement achieved by increasing the moving speed. Consider the detection time lag ratio for example in Figure 9a. The highest ratio or performance differential is around 2 when the sensor density is small. This means that by doubling the mobile sensor speed, the detection time lag in this region is almost halved. This verifies the assumption that for the same environment, it takes half the time to search for an anomaly with a doubled speed.
2. When comparing region 2 with region 1, the static sensor density is higher. The detection time ratio decreases to around 1.4 because the mobile sensor proportion β is small and the majority of fires are detected by the static sensors. As a result, the mobile sensor speed does not affect the monitoring performance as drastically as in region 1.
3. When comparing region 3 with region 1, the mobile sensor density is found to be higher. The detection time ratio decreases to around 1.6 because of the saturation effect. As discussed in Section 6.2, after a saturation point, the incremental improvement obtained from additional sensors decreases. For a similar reason, the improvement achieved by increasing the mobile sensor speed decreases when the mobile sensor density is high.
4. In region 4, both mobile sensor density and static sensor density are high. The performance ratio gradually plateaus, around and below 1.4.

In short, higher mobile sensor speed results in better monitoring performance, and that speed has a nonlinear effect on performance, which varies with sensor density and portfolio mix β. 

**Figure 10:**
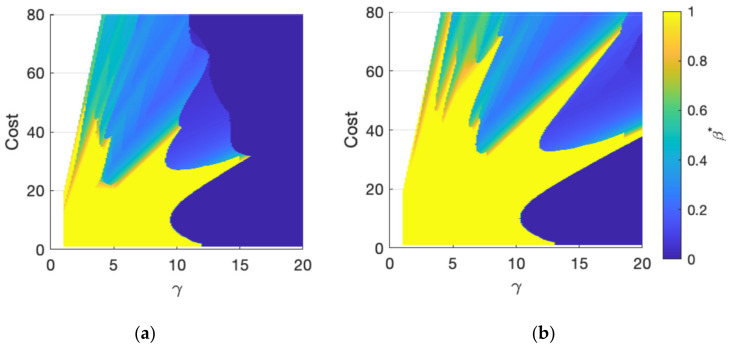
Optimal mobile sensor portfolio mix $\beta^{*}$ for different speeds: (**a**) 5 cm/s; (**b**) 20 cm/s.

The optimal portfolio mix $\beta^{*}$ also changes with the speed of mobile sensor. “Asymptotic thinking” helps to develop ideas about this observation. To illustrate this, assume first that the mobile sensor speed tends to 0, in which case, they provide a similar performance of the static sensors but at a higher cost (γ>1). They are therefore discarded in the optimization process, and the resulting optimal monitoring network will consist of purely static sensors $\left(\beta^{*}=0\right)$. On the other extreme, assume that the mobile sensor speed tends to infinity. In this hypothetical case, a single mobile sensor can be present at any location at any time and fulfill the function of an infinitely large static sensor network. Once the given budget allows for the acquisition of a single mobile sensor, the optimization process will switch to a purely mobile sensor network $\left(\beta^{*}=1\right)$. The reality will be somewhere between these two extremes, and the optimal portfolio mix $\beta^{*}$ will change in relation to the speed of the mobile sensor. The comparative results of $\beta^{*}$ in the C-γ space for mobile sensor speeds of 5 and 20 cm/s are provided in Figure 10. Only the detection time lag is included to avoid visual clutter.

The most significant result in Figure 10 is that the region in C-γ space where mobile sensor networks are optimal $\left(\beta^{*}=1\right)$ is significantly larger when the sensor speed is higher. It further expands/shrinks as the mobile sensor speed increases/decreases in accordance with the asymptotic thinking described previously.

Beyond these detailed results, it is useful to reflect on the main high-level findings this section demonstrated: (1) that a simulation-based analysis of sensor networks provides a more direct assessment of the ultimate objectives of specific monitoring and anomaly search tasks. It is myopic to restrict the analysis to indirect measures of the performance, such as area coverage; (2) that given specific cost parameters, hybrid sensor networks can provide significant and robust advantages over purely static or mobile sensor networks; (3) that identifying the optimal sensor portfolio mix requires careful analysis and is driven by cost parameters and mobile sensors speed.

The analyses and results provided here offer a fruitful basis for significant more developments in the future, and they raise an expansive set of additional questions briefly described in the conclusion.

## 7. Conclusions and Future Work

In this work, a collaborative static sensor allocation and mobile sensor path planning method was devised for the design and performance optimization of a hybrid sensor network (HSN). First, a weighted Gaussian coverage (WGC) method was designed for static sensor allocation, and the Active environmental Monitoring and Anomaly Search System (AMASS) was adapted to the path planning problem for the mobile sensors. Second, the optimal sensor portfolio mix was identified under different cost constraints. Third, the effect of mobile sensor speed on the network performance and optimal sensor portfolio was investigated. To analyze and validate the performance of the collaborative deployment method, a realistic simulation environment of fire searching in a 2D multi-room apartment was developed. The results demonstrate that: (1) a simulation-based analysis and the validation of sensor networks provide direct assessment of the ultimate objectives of specific monitoring and anomaly search tasks; (2) given specific cost parameters, collaborative hybrid sensor networks can provide significant advantages over either static or mobile sensor networks; (3) identifying the optimal sensor portfolio mix requires careful analysis and determined by cost parameters and mobile sensor speed.

This work addressed several research questions, and it raised a host of others, which point the way toward several future avenues for research. For example, the deployment strategy was developed for the purpose of detecting a single type of anomaly with a single type of sensor (single-anomaly-single-type-sensor). The examination of a heterogeneous suite of sensors, that monitor multiple states and detect multiple types of anomalies (multi-anomaly-multi-type-sensor), can be performed in future studies. Another important research direction involves not just the performance of the sensing network, but an analysis of its robustness and resilience in the face of different failure types or classes of threats. Such analyses help to further enrich an understanding of sensing network design trade space, and can further strengthen the appeal of hybrid sensor networks.

## Figures and Tables

**Table 1:** Design parameters for the hybrid sensor network problem.

Design Parameters	Descriptions
C	Total budget for the sensor network
$\gamma \in \left(1,\infty \right)$	Cost ratio of a mobile sensor over a static sensor
$n_{f}$	Number of static sensors
$n_{a}$	Number of mobile sensors
$N_{tot}\in \left[\frac{C}{\gamma },C\right]$	Total number of sensors (both static and mobile sensors)
$\beta \in \left[0,1\right]$	The proportion of mobile sensors in the network. Referred to as the portfolio mix

**Table 2:** Performance metrics in this application.

Performance Metrics	Descriptions
Detection time lag	The time to detect an anomaly after it occurs
Alarm distance	The distance between the alarmed sensor and the anomaly source
Estimation error	The median error between the estimated state value and the true measurement

**Figure A1:**
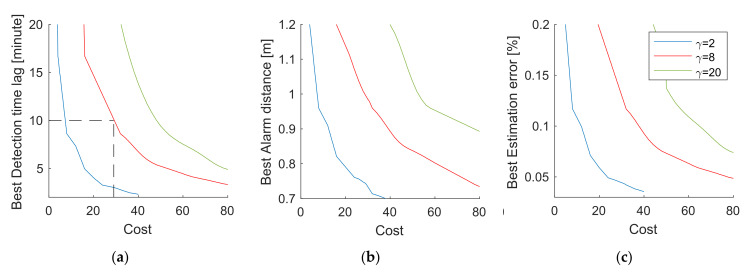
Performance-cost Pareto front of different γ: (**a**) Detection time lag; (**b**) Alarm distance; (**c**) Estimation error. The $n_{f}$-$n_{a}$ plane is divided into four regions (1–4). Larger γ represents current technology (expensive moving platforms). Smaller γ reflects the situation when moving platforms become more affordable. Note that the optimal portfolio mix $\beta^{*}$ varies along these Pareto fronts.

## Data Availability

Not applicable.

## Appendix A

### Dynamic Model of the Simulation Environment

A conduction heat propagation process has been adopted as given in Equation (A1) to simulate realistic indoor conditions [43]:

(A1) $$\frac{\partial T}{\partial t}=\alpha \nabla^{2}T+\frac{Q}{\rho c_{v}},$$

where t stands for time, T the temperature field, α the thermal diffusivity, ρ the air density,$\;c_{v}$ the constant volume specific heat constant, and Q the external volumetric heat release rate. The boundary condition is set to be adiabatic. Although a specific simulation environment has been developed for this study, other options are possible and users can adopt other models or high-fidelity simulation software, for example the Fire Dynamics Simulator (FDS) developed by the National Institute of Standards and Technology (NIST) [46], to obtain a suitable dynamic field for their specific applications.

The simulation parameters are selected to reflect a realistic situation. Air properties are taken at 300 K (27 ℃), and the heat release rate of the fire source is set to be 5000 W [47,48]. The probability of fires occurring at high-risk locations is set to be ten times as higher than the other locations. The complete simulation parameters are provided in Table A1.

**Table A1:** Performance metrics in this application.

Parameters	Descriptions	Value
κ	Heat conductivity	$0.02638\;\left[W\cdot m^{-1}K^{-1}\right]$ at 300 K
$c_{v}$	Specific heat capacity at constant volume	$0.718\;\left[\mathrm{kJ}\cdot {\mathrm{kg}}^{-1}K^{-1}\right]$ at 300 K
ρ	Density	$1.161\;\left[\mathrm{kg}\cdot m^{-3}\right]$ at 300 K
α	Thermal diffusivity	$22.63\times 10^{-6}\;\left[m^{2}\cdot s^{-1}\right]$ at 300 K
HRR	Heat release rate	$5000\;\left[W\right]$
$T_{a}$	Alarm temperature	$47\;\left[℃\right]$

## Appendix B

### Performance-Cost Pareto Fronts

The previous results, presented in Figure 7, identify the optimal sensor portfolio mix under various cost conditions in the C-γ space. However, what happens in instances where the optimal portfolio mix is no longer the primary focus, and the interest lies instead in identifying the best monitoring performance that can be achieved for a given budget C? This performance–cost multi-objective optimization problem is considered next, and the performance–cost Pareto fronts are identified. This analysis can help to inform several decisions, for example, to determine the required cost to meet a minimum performance requirement (e.g., a detection time lag of less than 10 min); or to assess whether a small increase in C can help “buy” a significant improvement in monitoring performance or not. To help address these decision problems, the performance–cost Pareto fronts for different γ are provided in Figure A1.

Figure A1 illustrates that the Pareto front with smaller γ dominates those with a larger γ Pareto front. Cheaper mobile sensors are favorable for their mobility and flexibility, and they can provide better performance. For the same cost, better monitoring performance is achieved with smaller γ. This reflects the changes in the optimal portfolio mix with the addition of mobile sensors. Moreover, it can be directly read from the Pareto front the network cost required to meet some performance requirement. For example, when γ=8, a detection time lag of less than 10 min requires a cost of around 30 (red curve and dashed lines in Figure A1a). Furthermore, the Pareto front reveals whether it is worth increasing the budget for the sensor network. For example, when γ=8, an increase in C from 20 to 25 can lead to considerable performance improvements for the detection time lag (and other performance metrics), whereas an increase from 60 to 65 will result in a minor improvement.
